# Timing is everything: priority effects alter community invasibility after disturbance

**DOI:** 10.1002/ece3.940

**Published:** 2014-01-20

**Authors:** Celia C Symons, Shelley E Arnott

**Affiliations:** Department of Biology, Queen's UniversityKingston, ON, Canada

**Keywords:** Experimental introduction, fluctuating resource hypothesis, freshwater ponds, metacommunity, nutrient addition, resource availability, resources, salinity

## Abstract

Theory suggests that communities should be more open to the establishment of regional species following disturbance because disturbance may make more resources available to dispersers. However, after an initial period of high invasibility, growth of the resident community may lead to the monopolization of local resources and decreased probability of successful colonist establishment. During press disturbances (i.e., directional environmental change), it remains unclear what effect regional dispersal will have on local community structure if the establishment of later arriving species is affected by early arriving species (i.e., if priority effects are important). To determine the relationship between time-since-disturbance and invasibility, we conducted a fully factorial field mesocosm experiment that exposed tundra zooplankton communities to two emerging stressors – nutrient and salt addition, and manipulated the arrival timing of regional dispersers. Our results demonstrate that invasibility decreases with increasing time-since-disturbance as abundance (nutrient treatments) or species richness (salt treatments) increases in the resident community. Results suggest that the relative timing of dispersal and environmental change will modify the importance of priority effects in determining species composition after a press disturbance.

## Introduction

Priority effects, where early arriving species affect the establishment of later arriving species, influence community assembly in new communities (e.g., Shulman et al. 1983; Louette and De Meester 2007; Körner et al. 2008) and community reassembly after disturbance (e.g., Mergeay et al. 2011). Priority effects are often attributed to niche preemption, when early arriving species are able to monopolize resources and gain a numerical advantage before the arrival of later dispersers (De Meester et al. 2002). The timing of dispersal can modify the importance of priority effects, with longer times between dispersal events increasing the dominance of early arriving species (Körner et al. 2008; Kardol et al. 2013). Additionally, environmental context can influence importance of arrival timing, with priority effects being stronger in productive environments than in resource-limited situations because species are able to preempt resources more quickly (Chase 2010; Kardol et al. 2013).

The importance of priority effects in the reassembly of communities after disturbances is likely influenced by the impact the disturbance has on resources (Davis et al. 2000). This is summarized by the fluctuating resource hypothesis of invasibility, which suggests that invasibility is positively related to the amount of unused resources that can be used by arriving species (Davis et al. 2000). Disturbance can either increase or decrease available resources (Sher and Hyatt 1999). The amount of unused resources can increase in two ways: resource availability can increase or resource use by the resident community can decrease (Davis et al. 2000). Experiments that have manipulated abiotic and/or biotic variables and controlled propagule pressure have shown that disturbance increases invasibility in terrestrial systems (e.g., Burke and Grime 1996; Davis and Pelsor 2001), freshwater systems (Strecker and Arnott 2010 *but see* Forrest and Arnott 2006), and marine systems (e.g., Clark and Johnston 2009).

The fluctuating resource hypothesis emphasizes that community invasibility changes through time (Davis et al. 2000). After a disturbance, resources can be used by the remaining local community, leading to a priority effect (De Meester et al. 2002; Urban and De Meester 2009). For example, after the chemical recovery of acidified lakes, there is evidence that interactions with the resident community limit the re-establishment of some acid-sensitive species, resulting in a lag in biological recovery (Binks et al. 2005). However, depending on whether the disturbance is directly providing resources, or reducing resource uptake by the local community, there may be differences in the importance of priority effects in community reassembly. If the disturbance is directly providing resources, then priority effects will likely be important, with the resident community monopolizing the new resources (De Meester et al. 2002; Urban and De Meester 2009; Chase 2010). Conversely, if the disturbance is lethal and reduces resource uptake by the local community, then priority effects may not be as important postdisturbance and communities may be more reliant on the arrival of tolerant species to utilize available resources. This may cause communities disturbed by a stressor that decreases resource uptake to be invasible for a longer period of time after disturbance than communities impacted by stressors that directly increase resources; however, this question has not been tested.

It is unclear how dispersing species will interact with resident species as global environmental conditions change. A region predicted to be disproportionately affected by climate change is the Subarctic. Under current climate change models, higher temperatures are expected to increase nutrient loading and evaporation, leading to higher nutrient concentrations and salinity in aquatic systems (Rautio et al. 2011). Additionally, large and increasing populations of snow geese (*Chen caerulescens caerulescens*) are found in this region (Cooke et al. 1995); snow geese have been shown to increase nutrients and conductivity of tundra ponds (Milakovic et al. 2001; Van Geest et al. 2007). Therefore, increasing nutrients and salts are expected to occur across two different temporal and spatial scales in subarctic regions.

For this study, we conducted a field experiment in Canada's Subarctic to determine whether two constant (i.e., press) disturbances, increases in nutrient and salt concentration, could influence the invasibility of zooplankton communities in tundra pond ecosystems. Increasing nutrients represent a disturbance that increases resources, and increasing salinity represents a disturbance that decreases resource uptake by the resident community because zooplankton have low tolerance to increased salinity resulting in a lower metabolism or death (Nielsen et al. 2003). We added dispersers at three different times postdisturbance to investigate how invasibility changes through time and test whether the importance of priority effects in community reassembly is different between the two types of disturbance.

We predicted that (i) the invasibility of zooplankton communities would be higher in disturbed communities than undisturbed communities, (ii) the invasibility of communities would decrease through time after disturbance, and (iii) the invasibility of high-salinity communities would remain high for a longer period of time postdisturbance than high-nutrient communities. Here, we report results suggesting invasibility declines after environmental change as priority effects influence the establishment success of regional dispersers.

## Materials and Methods

To determine whether environmental change and dispersal timing can modify the invasibility of zooplankton communities, we conducted an enclosure experiment where environmental conditions and dispersal were manipulated. The experiment was conducted in Golf Lake (58.7531 N,-93.9680 W) between 26th June and 16th August 2011. Golf Lake is an 11.4 ha, 1.5-m-deep pond located near Churchill, Manitoba, 2 km from the coast of Hudson Bay.

Clear polyethelene enclosures 50 cm in diameter, 80 cm deep with a total volume of 157 L (Filmtech Plastics, Brampton, ON, Canada) were suspended from floating wooden frames anchored in the pond. Lake water added to the enclosures was filtered through 50-*μ*m mesh to remove crustacean zooplankton. The enclosures were covered with clear 4-mil plastic to reduce evaporation and prevent incidental aerial colonization. Resident zooplankton were collected from Golf Lake on 24th June 2011 with a 80-*μ*m mesh conical net with a diameter of 35 cm and added to each of the 48 experimental enclosures at ambient pond zooplankton density (mean 6.8 individuals per L). Four additional aliquots were condensed to 100 mL preserved in 70% ethanol for later enumeration. Analysis of variance on the zooplankton samples taken from each enclosure on June 30 confirmed that there were no pretreatment differences in zooplankton communities (abundance, Shannon–Weiner diversity, evenness, species richness, correspondence analysis axis 1 and 2 scores).

To examine invasibility, we conducted a 2 × 2 × 3 factorial experiment with nutrients (no nutrients, +nutrients), salinity (no salt, +salt), and dispersal time (regional dispersers added 5, 14 or 23 days after environmental manipulation) as factors. Nutrient and salinity treatments were established at the same time. Dispersers were added 5, 14, and 23 days after nutrient and salt addition, and hereafter, these treatments are referred to as short delay, medium delay and long delay, respectively. This design resulted in 12 treatments, each replicated four times.

Nutrient and salinity treatments were established on 27th June 2011 (day 0). Nitrogen and phosphorus (as NH_4_Cl and KH_2_PO_4_) were added to the +nutrient enclosures at a concentration of 50 *μ*g/L P and a N:P ratio of 7:1 by mass. A phosphorus increase in this magnitude was used because similar nutrient concentrations have been documented in ponds disturbed by snow geese (Van Geest et al. 2007). Nutrients were added to the +nutrient enclosures throughout the experiment every 9 days to maintain this level of enrichment, by adding the same amount of nutrients every 9 days, calculated by assuming a 5% loss of nutrients to mesocosm walls per day (Downing et al. 2008). Salinity treatments consisted of increasing conductivity to 4000 *μ*S/cm, which is within the range of conductivities of ponds disturbed by snow geese (Figure S1), and correspond to a conductivity above the LC50 of some species of zooplankton in the Churchill area (Jones 2012). NaCl, MgSO_4_, KCl, and MgCl_2_ were added at a Cl:Na:Mg:SO_4_:K ratio of 34:18:6:3:1 by weight, the average ratio of salts in ponds in the Churchill region based on 2009 data (S. Arnott, unpubl. data).

Regional dispersers were collected from 47 to 51 rock pools and ponds (four had dried for day 24 collections) within 4 km of Golf Lake on 2nd July, 11th July, and 20th July 2011 for the addition of dispersers in the short-, medium-and long-delay treatments, respectively. The 51 sites had a wide range of chlorophyll-*a* (Chl-*a*) concentrations (0.1–24.6, mean 2.9 *μ*g/L) and conductivities (14–21780, mean 3531 *μ*S/cm). To collect dispersers, 2 L of water was collected from each site and then condensed into individual 100-mL jars that were stored in a cooler with ice packs. Collected zooplankton were mixed together in 50-*μ*m-filtered Golf Lake water and added to the +dispersal enclosures at a concentration of 1% ambient density based on the total volume of water collected from the disperser sites. All sampling and zooplankton introduction occurred within 2 h. Four additional dispersal aliquots were preserved in 70% ethanol for later enumeration. Due to natural variability in the regional species pool, our dispersal inoculation changed through time. Although there were no differences in most community indices (see below), there was a higher abundance of dispersers collected for the short-delay treatment than the medium-delay treatment (ANOVA linear contrasts, *P* < 0.05).

### Sampling protocols

Enclosures were sampled before dispersers were added, 2 days following the addition of the resident zooplankton community of Golf Lake and every 9 days from the start of the experiment for 28 days. Chl-*a*, temperature, and conductivity were measured in each enclosure and in Golf Lake using a YSI 600OMS (YSI Incorporated, Yellow Springs, OH).

Zooplankton were sampled from each enclosure with an 8-cm-diameter tube sampler. Three 3 L samples were taken from locations chosen haphazardly in each enclosure and condensed on an 80-*μ*m mesh filter. These samples were pooled for a final sampled volume of 9 L, or 5.7% of the enclosure volume. The tube sampler was thoroughly rinsed between each enclosure. On the last sample date of each dispersal time treatment, the enclosures were sampled more extensively with a 60-L (38%) sample volume. Zooplankton were preserved in 70% ethanol for later enumeration.

All crustacean zooplankton were counted and identified on a Nikon SMZ800 stereomicroscope (Nikon Corporation, Tokyo, Japan). Zooplankton were identified to species except for chydorids (identified to genus) and ostracods due to uncertainty in identification. Taxonomic keys used include Ward and Whipple (1959) and Dodson et al. (2010).

### Calculations and statistical analysis

We tested for a nutrient effect on Chl-*a* concentration using Chl-*a* data from 30 June 2011, 3 days after the addition of nutrients, using a two-factor ANOVA with nutrients and salt as factors. Chl-*a* data were log-transformed to improve normality and prevent unequal variance among treatments.

### The effect of disturbance and dispersal timing on invasibility

For each enclosure, we calculated invasibility. First, we identified dispersers, defined as species that were present in the regional dispersal pool and absent from the local community (day 0). Invasion success (i.e., invasibility) was calculated as the relative abundance of dispersers in the community 28 days after the addition of dispersers. We also calculated the absolute abundance of dispersers as another measure of invasibility. A three-way ANOVA was used to determine the additive and interactive effects of salt, nutrients and dispersal time on invasibility. To further clarify the effect of dispersal time, a two-way ANOVA was used to determine the additive and interactive effects of nutrients and salt on invasibility at each dispersal time. A search for the minimum adequate model was conducted following Crawley (2005), starting with a fully factorial model. For each step of model simplification, a high-order term was dropped from the model and ANOVA comparisons were used to compare nested models. Terms were removed if the simpler model did not have statistically higher deviance than the original model (*P *> 0.05). Model simplification stopped when all terms were significant, or terms were included in a significant interaction.

### Potential mechanisms influencing invasibility

Theoretical work has consistently predicted a negative relationship between resident community diversity and invasibility (Levine and D'Antonio 1999), and invasibility is expected to increase with resource availability (Davis et al. 2000). To test for a diversity–invasibility and resource–invasibility relationships, a permutation ANOVA (due to non-normal distribution of data) was used to test for an effect of Shannon–Weiner diversity of the resident community and Chl-*a* at the time dispersers were added on community invasibility across treatments. Similarly, we conducted the same permutation ANOVA using species richness as the diversity measure. A permutation Pearson correlation test was used to investigate the diversity–invasibility and resource–invasibility relationships separately to graphically represent these relationships independent of treatments.

To test for evidence of the fluctuating resource hypothesis, we conducted a permutation ANOVA to test whether invasibility was related to the abundance of the resident community and the Chl-*a* concentration at the time dispersers were introduced.

To determine why invasibility varied among the three different dispersal times, differences in the resident zooplankton community, regional dispersers, and environmental conditions were investigated using *a priori* linear contrasts as described below.

Differences in the resident community among the short-delay, medium-delay, and long-delay treatments were investigated using ANOVA *a priori* linear contrasts. Species richness, Shannon–Weiner diversity, evenness (*E*_var_), and abundance of the resident community at the time dispersers were added were compared among three dispersal times for each nutrient and salt treatment level (Quinn and Keough 2002). The *P*-values were corrected for multiple comparisons using the Benjamini–Hochberg false discovery rate (FDR) correction (Benjamini and Hochberg 1995).

Changes in the regional disperser pool between the short-delay, medium-delay, and long-delay treatments were investigated by comparing species richness, diversity, evenness, abundance, correspondence analysis axis 1, and 2 scores among the three dispersal times using ANOVA.

Finally, to determine whether environmental conditions varied among the short-delay, medium-delay, and long-delay treatments, the average and initial temperature, conductivity and chl-*a* were compared among the three dispersal times for each treatment level of salt and nutrients using ANOVA *a priori* linear contrasts (Quinn and Keough 2002). The *P*-values were corrected for multiple comparisons using the Benjamini–Hochberg FDR correction (Benjamini and Hochberg 1995).

### Composition changes in resident communities

We used correspondence analysis (CA), a unimodal ordination technique, to determine how disturbance and dispersal influenced zooplankton community composition. Data from all enclosures from each sample date were included to determine how community composition changed through time. Disperser samples were also included in the CA. A unimodal ordination technique was chosen because gradient lengths were >3 (Quinn and Keough 2002), which were assessed using a detrended correspondence analysis in CANOCO 4.5 (DCA, gradient length = 5.6). Extremely rare species (occurrence: <5%, percentage of total abundance: <0.02%) were removed from this analysis as they can have a disproportionate influence on ordination results (Quinn and Keough 2002). Remaining species abundances were Hellinger-transformed to reduce the influence of rare species and zeros, which are typically high in community data (Legendre and Gallagher 2001). To generate ordination diagrams, site scores were averaged between treatment replicates and plotted.

All analyses were completed in R 2.14.0 (R Development Core Team 2011) unless otherwise noted, using packages “vegan” (Oksanen et al. 2012) and “nlme” (Pinheiro et al. 2011).

## Results

Following the establishment of the nutrient treatments, there was an increase in chl-*a* concentration in the +N enclosures, and a decrease in chl-*a* in +S enclosures (nutrients: *F*_1,64_ = 128, *P *< 0.001, Salt: *F*_1,64_ = 13, *P *< 0.001).

### The effect of disturbance and dispersal timing on invasibility

The results of an analysis of variance revealed that invasibility of salt treatments changed with dispersal time (ANOVA, S*D: *F*_1,38_ = 5.99, *P* = 0.006) and the effect of salt depended on the nutrient treatment (ANOVA, S*N: *F*_2,38_ = 5.45, *P* = 0.025). Because we were particularly interested in determining how invasibility of individual treatments changed with dispersal time, we completed an ANOVA on each dispersal time separately.

**Figure 1:**
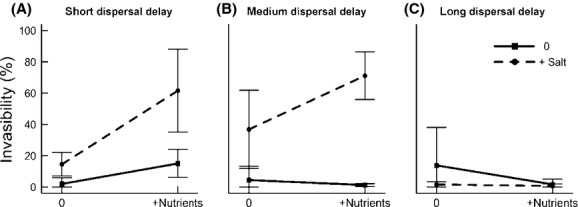
The invasibility of zooplankton communities in the salt and nutrient treatments for the (A) short-delay, (B) medium-delay and (C) long-delay dispersal treatments. The vertical lines represent standard deviation of the means.

The invasibility in both the salt and nutrient treatments decreased as the time between disturbance and dispersal increased, but the decrease was faster in the nutrient treatment (Fig. 1). The results of the two-way ANOVAs reveal that in the short-delay treatment, invasibility of the +S and +N communities was higher than the unmanipulated control (Fig. 1A; Table 1). In the medium-delay treatment, the +S communities were still more invasible than the controls, but there was no longer a difference in invasibility between the +N and control communities (Fig. 1B; Table 1). Finally, in the long-delay treatments, invasibility was low for all treatments and no differences in invasibility were detected (Fig. 1C; Table 1). When investigating the individual dispersal times all nutrient and salt treatment effects were additive, and there was no evidence of a N*S interaction. The results of this analysis are very similar if invasibility is defined as the abundance of invaders instead of relative abundance. The only difference is that when dispersers are added 14 days after disturbance there is an interaction between salt and nutrients, where salt increases invasibility, and invasibility is highest when salt and nutrients are added together (ANOVA, *F* = 27.3, *P *< 0.001).

**Table 1:** Results of the minimum adequate ANOVA models conducted to determine whether invasibility differed significantly between nutrient and salt treatments at each dispersal time. Degrees of freedom in the short-delay treatments are *F*_1,13_, and in the medium-delay treatments are *F*_1,14_ for the minimum adequate ANOVA model.

	Nutrients		Salt		N^*^S	
	*F*	*P*	*F*	*P*	*F*	*P*
Short delay	31.3	<0.001	30.8	<0.001	–	–
Medium delay	–	–	44.9	<0.001	–	–
Long delay	–	–	–	–	–	–

### Potential mechanisms influencing invasibility

**Figure 2:**
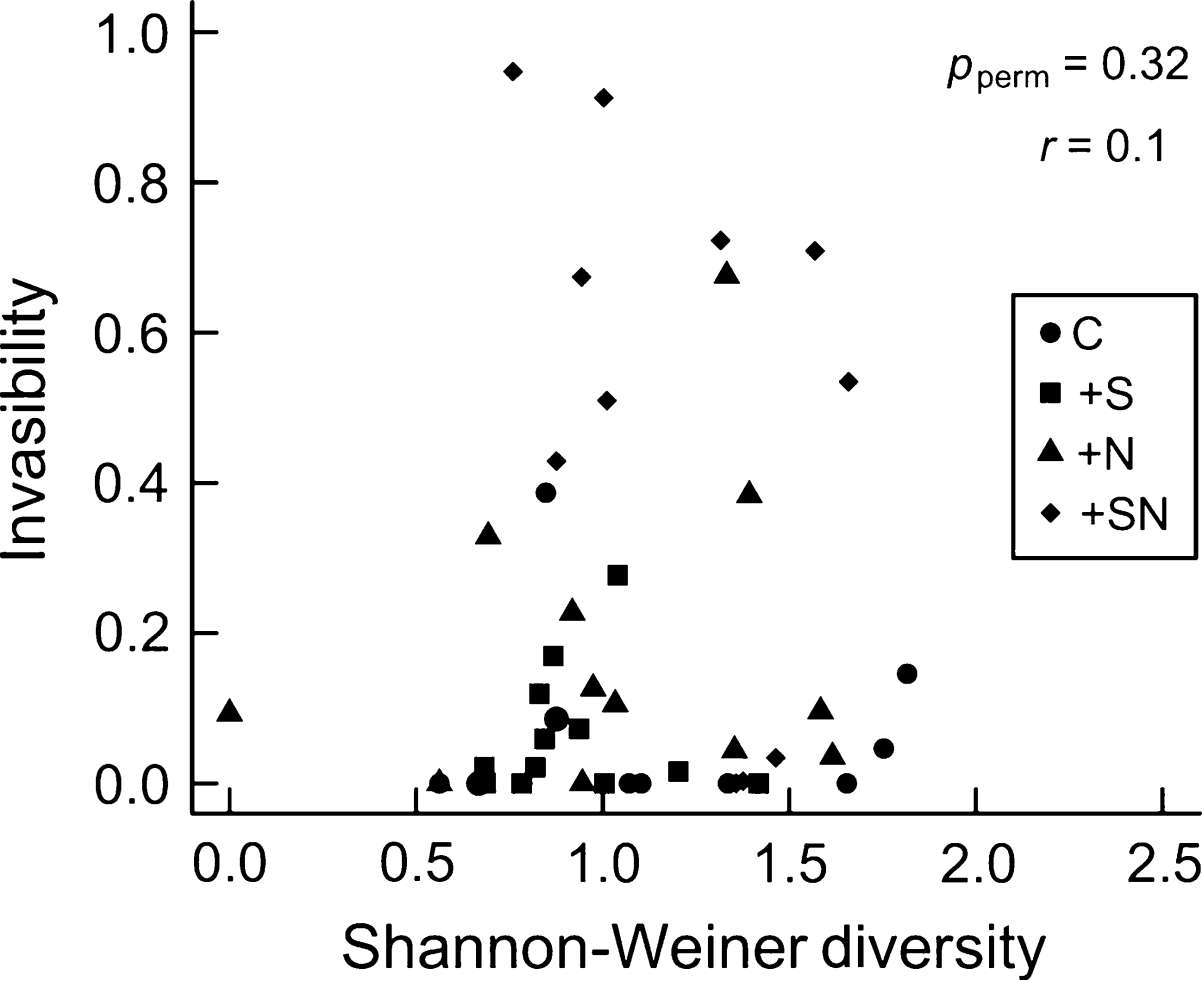
The relationship between the diversity of the community when dispersers were added and invasibility in the control (C), +nutrient (+N), +salt (+S), and salt+nutrient (+SN) treatments.

**Figure 3:**
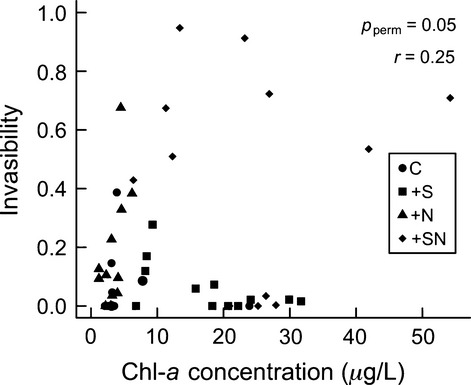
The relationship between the chl-a concentration (proxy for resource availability) in the enclosures when dispersers were added and invasibility in the control (C), +nutrient (+N), +salt (+S), and salt+nutrient (+SN) treatments.

We tested for evidence of diversity–invasibility and resource–invasibility relationships. We found that there was no relationship between the diversity of the resident community and its invasibility, but invasibility increased with Chl-*a* concentration (permutation ANOVA, diversity *P*_perm_ = 0.48, Chl-*a P*_perm_ = 0.053; Figs 2 and 3). Similar results were obtained when using species richness in place of Shannon–Weiner diversity (permutation ANOVA, richness *P*_perm_ = 0.92, Chl-*a P*_perm_ = 0.03).

**Figure 4:**
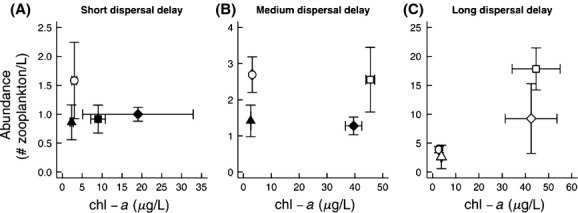
The chl-*a* concentration (resource supply) and zooplankton abundance (resource uptake) for each treatment on the day dispersers were added for the (A) short-delay, (B) medium-delay, and (C) long-delay treatments. Circles represent control enclosures, squares represent +nutrient enclosures, triangles represent +salt enclosures, and diamonds represent salt+nutrient enclosures. Filled symbols are more invasible than the control, as determined by one-way ANOVAs (Fig. 1). The error bars represent standard error. Note different scales on the y-axis for each panel.

We found that our results provide support for the fluctuating resource hypothesis of invasibility because both the abundance of the resident community and concentration of Chl-*a* at the time dispersers were introduced influenced invasibility (permutation ANOVA, abundance *P*_perm_ = 0.03, Chl-*a P*_perm_ = 0.06). To more directly compare our results to the fluctuating resource hypothesis, we plotted the resident community abundance and chl-*a* concentration at the time dispersers were added – zooplankton abundance is a proxy for resource use, and chl-*a* is a proxy for resource supply (Fig. 4). Davis et al. (2000) suggest that a shift toward the bottom right in a plot of resource supply and resource use would result in increased invasibility because the amount of available resources for dispersers would increase. When dispersers were added 3 days after disturbance, the more invasible communities were located further to the bottom right than the controls, with lower abundance and higher chl-*a* concentration, in accordance with the fluctuating resource hypothesis (Fig. 4A). When dispersers were added 12 days after disturbance, we did not find this pattern – the +N treatment with high chl-*a* concentration was not more invasible than the control (Fig. 4B). Finally, when dispersers were added 24 days after disturbance, invasibility was low in all treatments, although there were differences in abundance and resource availability (Fig. 4C).

**Figure 5:**
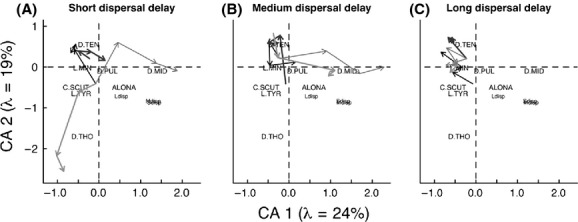
Biplot of CA scores for the zooplankton communities for the (A) short-delay, (B) medium-delay and (C) long-delay treatments. *λ* represents the amount of variation explained. The control treatment is plotted in black (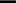), +nutrient treatment is thick dark gray (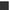), +salt treatment is this light gray (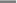), and +nutrients+salt treatment is thick light gray (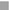). The arrows represent progression through time from day 1 to day 28. Species are denoted D.TEN, *Daphnia tenebrosa;* L.MIN, *Leptodiaptomus minutus*; C.SCUT, *Cyclops scutifer*; L.TYR, *Leptodiaptomus tyrelli;* D.PUL, *Daphnia pulicaria;* ALONA, *Alona* spp.; D.MID, *Daphnia middendorfianna;* and D.THO, *Diacyclops thomasi*. Golf Lake residents are L.MIN, D.TEN, C.SCUT, L.TYR, and ALONA. The dispersers added at each time are plotted as Sdisp, Mdisp, and Ldisp for the short-, medium-and long-delay treatments, respectively.

The resident community changed after the establishment of salt and nutrient treatments so that the resident community was different at the time of disperser addition for the short-delay, medium-delay and long-delay treatments potentially influencing community invasibility. When nutrients were added, the abundance of the resident community increased at each dispersal time relative to the previous dispersal time (ANOVA linear contrasts, *P *< 0.05; Tables S1 and S2). The nutrient treatments also caused a decrease in evenness through time (ANOVA linear contrasts, *P *< 0.05; Tables S1 and S2), as *Daphnia tenebrosa* G.O. Sars, a large grazer, became dominant in the resident community (Fig. 5). Species richness was higher in the +S and +SN treatments at the start of the medium delay than the short delay due to the maturation of copepods and the increase in abundance of species beyond detection limits (ANOVA linear contrasts, *P *< 0.05; Tables S1 and S2). Changes in the resident community were not detected in the control treatment – there were no significant differences in diversity, evenness, richness, and abundance among the short-, medium-and long-delay treatments (ANOVA linear contrasts, *P *> 0.05; Table S2).

When investigating whether changes in the disperser pool could influence invasibility, we found the abundance of dispersers between two of the dispersal times differed due to changes in the zooplankton communities in ponds from where dispersers were collected. There was a higher abundance of dispersers added in the short delay than the medium delay (ANOVA, *F*_2,9_ = 8.0, *P *= 0.01; Tukey's HSD test, *P *= 0.01), but no differences between the short and long delay or the medium and long delay (ANOVA, *F*_2,9_ = 8.0, *P *= 0.01; Tukey's HSD test, *P *> 0.05). Although the abundance changed, most aggregate community measures of the disperser pool did not differ between dispersal times, including Shannon–Weiner diversity, *E*_var_, richness, correspondence analysis axis 1 and 2 scores (Table S3).

The average environmental conditions were compared between the three dispersal times as another potential mechanism for changes in invasibility; however, the mean temperature, conductivity, and chl-*a* concentration that the dispersers experienced did not differ significantly among the 3 dispersal times. Water temperature on the day dispersers were added significantly decreased through time (ANOVA linear contrasts, Table S4).

### Composition of dispersers and resident communities

An average of 11 (±1.4 SD) novel species were added as dispersers at each dispersal time, and throughout all three dispersal times, we detected 13 novel species (Table S5). The disperser pool consisted of an average of 21 (±10 SD) copepods and 48 (±11 SD) cladocerans. Seven of the 13 novel species in the disperser pool successfully established in at least one enclosure by the end of the experiment. Six of the seven species that established were asexual cladocerans, and one was a sexual copepod. Six species did not establish, including four sexual copepods and two asexual cladocerans.

Zooplankton community composition changed in all treatments throughout the course of the experiment (Fig. 5). The CA axis 1 and 2 explained 24% and 19% of the variation in community composition, respectively. In the short-delay treatments, the control enclosures were dominated by *Daphnia tenebrosa* and *Leptodiaptomus minutus* (Lilljeborg), two regionally common species. In the +N enclosures, three *Daphnia* species became dominant: *Daphnia pulicaria*, *Daphia pulex,* and *Daphnia middendorffiana* (Fig. 5A). +S enclosures became dominated by the cyclopoid disperser *Diacyclops thomasi* (Forbes) and the +SN enclosures became dominated by the disperser *Daphnia middendorffiana* (Fischer; Fig. 5A). In the medium-delay treatments, the control treatment community composition remained associated with the resident community species. The +N enclosures were dominated by *D. tenebrosa,* and the +S and +SN treatments were dominated by the disperser *D. middendorffiana* (Fig. 5B). In the long-delay treatments, all communities were located in the bottom right corner of Figure 5C, associated with resident community species. The +N treatments were dominated by *D. tenebrosa,* and +S treatments were copepod-dominated (Fig. 5C).

## Discussion

The results of our enclosure experiment show that disturbance weakened the biotic resistance of communities, increasing invasibility. However, the press disturbances only increased invasibility for a short period of time, with the invasibility of the local communities decreasing through time after disturbance. Interestingly, the resident community reduced invasibility faster in the “resource-added” treatments (i.e., +nutrients) than the “reduction-in-resource-uptake” treatment (i.e., +salt). Few studies have investigated how invasibility changes after a press disturbance (but see James et al. 2006; Li and Stevens 2012), and to our knowledge, comparisons of disturbances that either add resources or cause a reduction in resource uptake and their temporal effect on invasibility have not been made.

### The effect of disturbance on invasibility

Disturbance increased invasibility due to higher availability of resources (i.e., chl-*a*) in the +nutrient enclosures, and lower resource uptake (i.e., abundance) in the +salt enclosures when dispersers were added shortly after environmental manipulations in accordance with the fluctuating resource hypothesis (Fig. 4A; Davis et al. 2000). In agreement with this, invasibility was highest in the salt+nutrient treatment where both nutrient supply increased and resource uptake decreased. Under control conditions, invasibility was low, and disperser species rarely established, or remained at low relative abundance. This supports the results of many aquatic and terrestrial experiments where undisturbed communities resist the establishment of dispersers (Shurin 2000; Fargione et al. 2003; Forrest and Arnott 2006; Strecker and Arnott 2010).

Theoretical models suggest that disturbance can increase invasibility by decreasing diversity, providing niche space for dispersers (Levine and D'Antonio 1999); however, neither diversity (Fig. 2) nor species richness was correlated with invasibility in this experiment (Fig. 2). We found that invasibility increased with resource availability (chl-*a*), suggesting that resource supply, independent of diversity, influenced community invasibility (Fig. 3). This agrees with experiments that have manipulated diversity and resources independently and found that resources were the main determinant of invasibility (e.g., Davis et al. 2000; Stachowicz et al. 2002). High residual variation in the relationship between chl-*a* and invasibility (Fig. 3) may have resulted from an interaction between the environmental treatments, for example salt treatments always had lower chl-*a* concentrations than nutrient treatments.

### Invasibility changed with time-since-disturbance

In agreement with the fluctuating resource hypothesis, invasibility was greatest after disturbance (Fig. 4A). However, when there was a longer delay between disturbance and dispersal, a higher concentration of chl-*a* (i.e., increased resource supply) no longer increased community invasibility (Fig. 4), providing a very short “window of opportunity” for dispersers to establish. This is in agreement with studies of plant and coral reef community assembly, which show that when there are longer delays between the arrival of species, the early arriving species exert stronger priority effects (Körner et al. 2008; Geange and Stier 2009; Kardol et al. 2013). Additionally, the importance of priority effects during community assembly is larger in high-resource environments than low-resource environments (Kardol et al. 2013). Consistent with this, we found that the lethal disturbance – salt addition – increased the invasibility of communities for a longer time after environmental manipulation than the resources-added disturbance, suggesting that priority effects were more important when nutrients were added (Fig. 1). Invasibility decreased in the nutrient treatments as the resident community abundance increased (Fig. 4) with *Daphnia tenebrosa* dominating the community by the time the medium-and long-delay treatments started (Fig. 5). This suggests that the resident community was able to exert strong priority effects by gaining a numerical advantage over less abundant disperser species, leading to niche preemption. Additionally, the lethal disturbance caused a reduction in initial community abundance (Fig. 4) and species richness, therefore reducing the number of individuals present to induce priority effects, which likely contributed to the different time scale of priority effects observed in the salt treatment (Fig. 1).

Priority effects can reduce invasibility through numerical effects, where rapid population growth allows resident species to reach habitat carrying capacity before dispersing species arrive (De Meester et al. 2002). The strength of priority effects is determined by the time it takes resident communities to monopolize resources and increase in abundance relative to the time it takes dispersers to arrive (Shulman et al. 1983). Many cladocerans, such as *D. tenebrosa*, have life cycles that promote priority effects, as they have high intrinsic capacities of population growth (De Meester et al. 2002). Cladocerans dominated nutrient treatments that likely strengthened priority effects relative to salt treatments, which were dominated by copepod species (Fig. 5) that have a longer life cycle and may not be able to monopolize resources as quickly as cladocerans (Thorp and Covich 1991). In general, salt stress reduces reproductive rates due to energy allocation to osmoregulation (Hart et al. 2003). With lower reproductive output, the resident community may require more time to monopolize resources and reduce invasibility after environmental change.

Invasibility can also be influenced by characteristics of the dispersal pool (e.g., propagule pressure, tolerance of the dispersers, reproduction rate, etc.). Propagule pressure is positively related to invasibility, due to reduced chance of stochastic extinction of dispersers (Pimm et al. 1988). The species that were able to establish during this experiment had the highest abundances in the disperser pool, suggesting that propagule pressure influenced the invasiveness of each species. Additionally, there was variation in the total abundance of dispersers that were added at each dispersal time (Table S3). There were more dispersers introduced in the short-delay treatment (mean: 79 individuals) than the medium-delay (mean: 58 individuals) treatment because there was a higher abundance in the disperser pools/ponds on the first sampling date. This may have contributed to the high invasibility seen in the short-delay treatments compared with the medium-delay treatments by reducing the likelihood of stochastic extinction of the dispersers; however, the abundance of dispersers added in the short-delay and long-delay treatments was similar (short delay: mean 79 individuals, long-term delay: mean 71 individuals) and invasibility decreased through time (Fig. 1), suggesting that propagule pressure was not likely the main driver influencing the change in invasibility through time.

Invasibility of the communities experiencing disturbance decreased through time such that 24 days after the disturbance, invasibility had decreased to the low levels characteristic of the undisturbed communities (Fig. 1C). Our study demonstrates that community reassembly after disturbance will, in part, be determined by species dispersal timing relative to the disturbance. How this ultimately influences community response to environmental change over the long-term is uncertain. One potential implication of low invasibility is a reduction in species richness (Shulman et al. 1983).

## Conclusions and Future Directions

Invasibility was measured after establishment, a key stage in population growth with early establishment often predicting long-term species patterns (Davis and Pelsor 2001; Foster and Tilman 2003). Our results suggest that dispersal timing will have significant effects on the response of communities to environmental change. If dispersal timing coincides with changing nutrient and salt concentrations – such as the arrival of snow geese to the Hudson Bay lowlands– then species composition may be able to track this change; however, if dispersal timing does not coincide with environmental change, then our results suggest that resident communities will monopolize resources and resist the establishment of new species. As the Arctic and Subarctic experience environmental change and increased development, understanding the factors controlling the establishment of new species including regional species and species dispersing long distances (e.g., species tracking new climate regimes, nonindigenous species, marine species invading inland lakes) is imperative. Limited information exists on factors controlling the timing of dispersal such as ephippial egg production and the movement of dispersal vectors. Our results highlight the importance of disturbance and dispersal timing in the invasion process, particularly when local species can readily monopolize free resources. The role of priority effects may make it difficult to predict species establishment after environmental change.

## Supporting Information

Table S1 Treatment mean and standard deviations (SD) at the beginning and end of the experiment for control (C), +nutrient (N), +salt (S), and +salt+nutrient (SN) treatments.

Table S2 Benjamini–Hochberg false discovery rate corrected *P*-values for ANOVA linear contrasts between the control (C), +nutrient (N), +salt (S), and +salt+nutrient (SN) treatments Benjamini–Hochberg false discovery rate corrected *P*-values for ANOVA linear contrasts between the control (C), +nutrient (N), +salt (S), and +salt+nutrient (SN) treatments.

Table S3 Results of one-way ANOVAs for the dispersers added at each dispersal-delay treatment to determine whether there were differences in the disperser pool throughout the season.

Table S4 Benjamini–Hochberg false discovery rate corrected *P*-values for ANOVA linear contrasts between the control (C), +nutrient (N), +salt (S), and +salt+nutrient (SN) treatments.

Table S5 Species that were detected in the disperser pool.

Figure S1 The conductivity of enclosures throughout the experiment and the conductivity of ponds disturbed by geese.
