# Supplementary material for: Timing is everything: priority effects alter community invasibility after disturbance
Source: Ecol Evol. 2014 Jan 20;4(4):397–407. doi: 10.1002/ece3.940 (PMC3936386; doi:10.1002/ece3.940)
Supplement: Table S1 — Treatment mean and standard deviations (SD) at the beginning and end of the experiment for control (C), +nutrient (N), +salt (S), and +salt+nutrient (SN) treatments. [file ece30004-0397-sd1.pdf]

Table S1. Treatment mean and standard deviations (sd) at the beginning and end of the experiment for control (C), +nutrient (N), +salt (S) and +salt+nutrient (SN) treatments. The three different dispersal-delay treatments are denoted as short, med and long for the 5, 14 and 23 delay between disturbance and dispersal respectively.

	C-short		C-med		C-long		N-short		N-med		N-long	
	mean	sd	mean	sd	mean	sd	mean	sd	mean	sd	mean	sd
Initial Diversity	0.69	0.13	1.35	0.35	1.40	0.41	0.92	0.09	1.03	0.34	0.83	0.13
Initial Evenness	0.71	0.16	0.65	0.30	0.61	0.45	0.74	0.22	0.84	0.12	0.71	0.28
Initial Species Richness	1.75	0.96	3.00	0.82	3.25	0.96	2.50	1.00	3.75	0.96	2.25	1.26
Initial Abundance	1.3	0.19	3.00	1.85	11.42	12.72	0.94	0.23	1.64	0.99	8.83	12.42
Initial Chl-*a*	4.38	2.29	8.20	10.47	3.00	0.75	11.12	5.01	26.6	5.10	15.78	6.54
Final Diversity	1.32	0.33	1.50	0.21	1.20	0.21	1.50	0.15	1.01	0.24	1.10	0.20
Final Evenness	0.73	0.23	0.81	0.16	0.76	0.24	0.36	0.11	0.35	0.30	0.24	0.19
Final Species Richness	3.00	0.82	4.00	1.15	2.50	1.29	5.25	0.96	3.25	0.96	2.25	0.50
Final Abundance	4.08	1.18	9.58	4.86	3.78	1.85	45.50	27.88	94.39	80.66	97.17	74.83
Final Chl-*a*	2.63	1.14	2.68	0.56	3.63	1.35	4.50	2.50	3.28	1.28	3.33	0.91

	S-short		S-med		S-long		SN-short		SN-med		SN-long	
	mean	sd	mean	sd	mean	sd	mean	sd	mean	sd	mean	sd
Initial Diversity	0.73	0.49	1.25	0.39	1.12	0.46	0.90	0.11	1.39	0.29	1.25	0.31
Initial Evenness	0.84	0.16	0.65	0.16	0.62	0.28	0.89	0.13	0.86	0.09	0.72	0.16
Initial Species Richness	2.00	1.15	3.25	1.26	2.75	0.50	1.75	0.50	2.50	1.29	3.75	0.96
Initial Abundance	1.58	1.32	1.39	0.46	8.75	7.20	0.53	0.42	1.92	0.88	4.59	2.87
Initial Chl-*a*	1.95	0.93	4.85	0.92	3.10	0.74	10.85	3.09	36.55	14.28	25.10	3.01
Final Diversity	1.43	0.13	1.91	0.26	1.00	0.24	1.49	0.33	1.33	0.31	1.40	0.11
Final Evenness	0.62	0.15	0.57	0.21	0.64	0.38	0.30	0.13	0.37	0.26	0.57	0.27
Final Species Richness	4.50	0.58	5.50	0.58	2.75	1.71	3.75	2.06	3.75	1.26	3.50	1.91
Final Abundance	11.11	1.79	6.31	1.08	8.86	5.61	37.14	37.03	50.28	25.59	38.28	38.43
Final Chl-*a*	7.05	4.27	3.55	1.36	2.83	0.40	10.90	7.74	7.03	2.97	4.70	2.60
