# Supplementary material for: Timing is everything: priority effects alter community invasibility after disturbance
Source: Ecol Evol. 2014 Jan 20;4(4):397–407. doi: 10.1002/ece3.940 (PMC3936386; doi:10.1002/ece3.940)
Supplement: Table S2 — Benjamini–Hochberg false discovery rate corrected P-values for ANOVA linear contrasts between the control (C), +nutrient (N), +salt (S), and +salt+nutrient (SN) treatments Benjamini–Hochberg false discovery rate corrected P-values for ANOVA linear contrasts between the control (C), +nutrient (N), +salt (S), and +salt+nutrient (SN) treatments. [file ece30004-0397-sd2.pdf]

Table S2. Benjamini-Hochberg false discovery rate corrected *p*-values for ANOVA linear contrasts between the control (C), +nutrient (N), +salt (S) and +salt+nutrient (SN) treatments. The three different dispersal-delay treatments are denoted as short, med and long for the 5, 14 and 23 delay between disturbance and dispersal respectively. Comparisons were made between the three dispersal-delay treatments for resident community initial and average Shannon-Weiner diversity, evenness (E_{var}), species richness and total zooplankton abundance

Treatments compared	C-short<br>C-med	C-short<br>C-long	C-med<br>C-long	N-short<br>N-med	N-short<br>N-long	N-med<br>N-long
Initial Diversity	0.29	0.29	0.99	0.71	0.80	0.35
Initial Evenness	0.89	0.20	0.51	0.03	0.03	0.13
Initial Species richness	0.10	0.56	0.56	0.17	0.41	0.63
Initial Abundance	0.85	0.23	0.59	0.001	<0.001	<0.001
Avg Diversity	0.87	0.87	0.88	0.87	0.87	0.87
Avg Evenness	0.98	0.76	0.76	0.37	0.37	0.43
Avg Species richness	0.79	0.79	0.79	0.79	0.79	0.79
Avg Abundance	0.94	0.90	0.94	0.33	0.33	0.37

Treatments compared	S-short<br>S-med	S-short<br>S-long	S-med<br>S-long	SN-short<br>SN-med	SN-short<br>SN-long	SN-med<br>SN-long
Initial Diversity	0.11	0.24	0.64	0.24	0.24	0.24
Initial Evenness	0.89	0.86	0.89	0.58	0.86	0.92
Initial Species richness	0.02	0.17	0.63	0.02	0.12	0.36
Initial Abundance	0.58	0.58	0.28	0.01	0.01	0.01
Avg Diversity	0.87	0.87	0.87	0.88	0.87	0.87
Avg Evenness	0.76	0.76	0.76	0.76	0.76	0.76
Avg Species richness	0.79	0.79	0.94	0.79	0.79	0.79
Avg Abundance	0.57	0.37	0.37	0.33	0.33	0.37
