# Supplementary material for: Timing is everything: priority effects alter community invasibility after disturbance
Source: Ecol Evol. 2014 Jan 20;4(4):397–407. doi: 10.1002/ece3.940 (PMC3936386; doi:10.1002/ece3.940)
Supplement: Table S3 — Results of one-way ANOVAs for the dispersers added at each dispersal-delay treatment to determine whether there were differences in the disperser pool throughout the season. [file ece30004-0397-sd3.pdf]

Table S3. Results of one-way ANOVAs for the dispersers added at each dispersal-delay treatment to determine if there were differences in the disperser pool throughout the season

Response variable	df	Sum of Squares	Mean Squares	F	*p*
Diversity	2	0.02	0.01	0.8	0.47
Residual	9	0.11	0.01		
Evenness	2	0.01	0.002	1.5	0.27
Residual	9	0.01	0.002		
Species richness	2	10.2	5.1	3.6	0.07
Residual	9	12.8	1.4		
CA1 scores	2	1.82	0.91	4.1	0.05
Residual	9	2.01	0.22		
CA2 scores	2	0.01	0.003	0.1	0.94
Residual	9	0.38	0.042		
Abundance	2	0.23	0.12	8.0	0.01
Residual	9	0.13	0.01		
