# Supplementary material for: Timing is everything: priority effects alter community invasibility after disturbance
Source: Ecol Evol. 2014 Jan 20;4(4):397–407. doi: 10.1002/ece3.940 (PMC3936386; doi:10.1002/ece3.940)
Supplement: Table S4 — Benjamini–Hochberg false discovery rate corrected P-values for ANOVA linear contrasts between the control (C), +nutrient (N), +salt (S), and +salt+nutrient (SN) treatments. [file ece30004-0397-sd4.pdf]

Table S4. Benjamini-Hochberg false discovery rate corrected *p*-values for ANOVA linear contrasts between the control (C), +nutrient (N), +salt (S) and +salt+nutrient (SN) treatments. The three different dispersal-delay treatments are denoted as short, med and long for the 5, 14 and 23 delay between disturbance and dispersal respectively. Comparisons were made between the three dispersal-delay treatments for initial and average Chl-*a* concentration (µg L^{-1}), temperature (C) and conductivity (µS cm^{-1})

Treatments compared	C-short C-med	C-short C-long	C-med C-long	N-short N-med	N-short N-long	N-med N-long
Initial Chl-*a*	0.98	0.98	0.98	0.08	0.09	0.42
Initial Temperature	<0.001	<0.001	<0.001	<0.001	<0.001	<0.001
Initial Conductivity	0.99	0.99	0.99	0.99	0.99	0.99
Ave Chl-*a*	0.99	0.99	0.99	0.99	0.99	0.99
Ave Temperature	0.09	0.13	0.09	0.50	0.75	0.16
Ave Conductivity	0.99	0.99	0.99	0.99	0.99	0.99

Treatments compared	S-short S-med	S-short S-long	S-med S-long	SN-short SN-med	SN-short SN-long	SN-med SN-long
Initial Chl-*a*	0.98	0.98	0.98	0.32	0.32	0.60
Initial Temperature	<0.001	<0.001	<0.001	<0.001	<0.001	<0.001
Initial Conductivity	0.99	0.99	0.99	0.99	0.99	0.99
Ave Chl-*a*	0.99	0.99	0.99	0.99	0.99	0.99
Ave Temperature	0.69	0.55	0.09	0.16	0.55	0.99
Ave Conductivity	0.99	0.99	0.99	0.99	0.99	0.99
