# Supplementary material for: Timing is everything: priority effects alter community invasibility after disturbance
Source: Ecol Evol. 2014 Jan 20;4(4):397–407. doi: 10.1002/ece3.940 (PMC3936386; doi:10.1002/ece3.940)
Supplement: Table S5 — Species that were detected in the disperser pool. [file ece30004-0397-sd5.pdf]

Table S5. Species added at each time step. The presence of a species at each time dispersers were added is denoted Y if it was present in an enclosure at the end of the experiment and N if it was in the disperser pool but failed to establish.

Species	Functional group	Reproduction	Dispersal delay		
			Short	Medium	Long
Ceriodaphnia laticaudata	Cladoceran	Cyclic parthenogen	Y	Y	-
Daphnia middendorffiana	Cladoceran	Obligate parthenogen	Y	Y	Y
Daphnia pulex	Cladoceran	Obligate parthenogen	Y	N	N
Daphnia pulicaria	Cladoceran	Cyclic parthenogen	N	Y	N
Diacyclops thomasi	Cyclopoid copepod	Sexual	Y	Y	Y
Polyphemus pediculus	Cladoceran	Cyclic parthenogen	N	N	Y
Scapholeberis kingii	Cladoceran	Cyclic parthenogen	N	N	Y
Diacyclops arcticus	Calanoid copepod	Sexual	N	N	N
Diaphanosoma brachyurum	Cladoceran	Cyclic parthenogen	-	N	N
Epischura lacustris	Calanoid copepod	Sexual	N	-	N
Eurytemora affinis	Calanoid copepod	Sexual	N	N	-
Microcyclops rubellus	Cyclopoid copepod	Sexual	N	N	-
Sida crystallina	Cladoceran	Cyclic parthenogen	-	-	N
