# Supplementary material for: Timing is everything: priority effects alter community invasibility after disturbance
Source: Ecol Evol. 2014 Jan 20;4(4):397–407. doi: 10.1002/ece3.940 (PMC3936386; doi:10.1002/ece3.940)
Supplement: Figure S1 — The conductivity of enclosures throughout the experiment and the conductivity of ponds disturbed by geese. [file ece30004-0397-sd6.pdf]

A

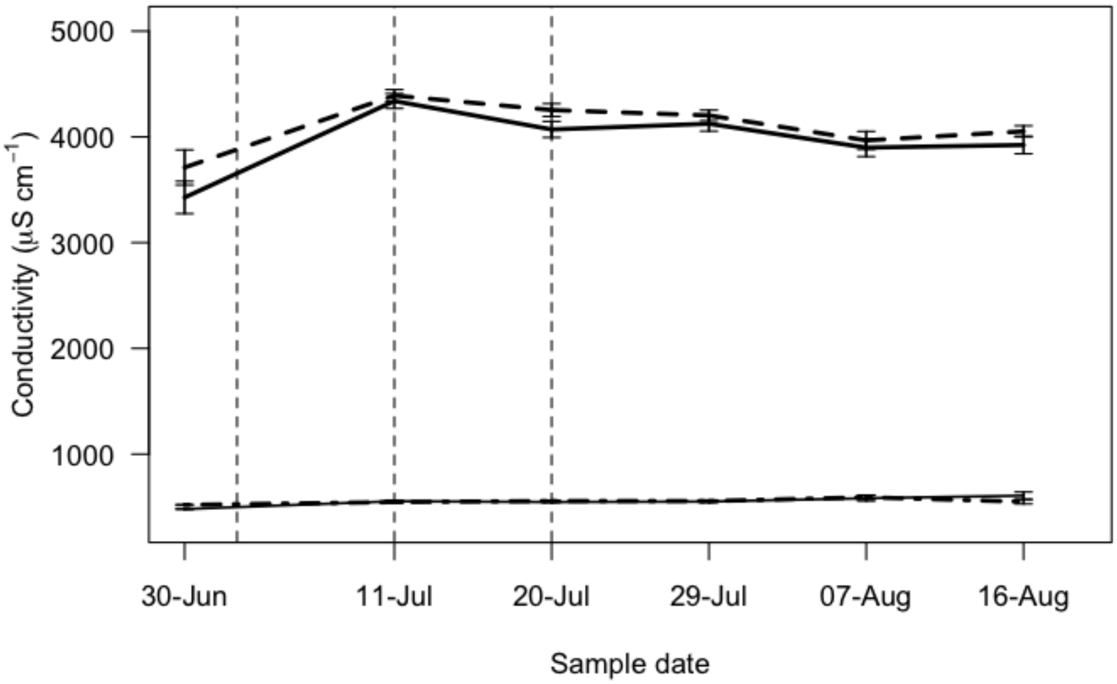

B

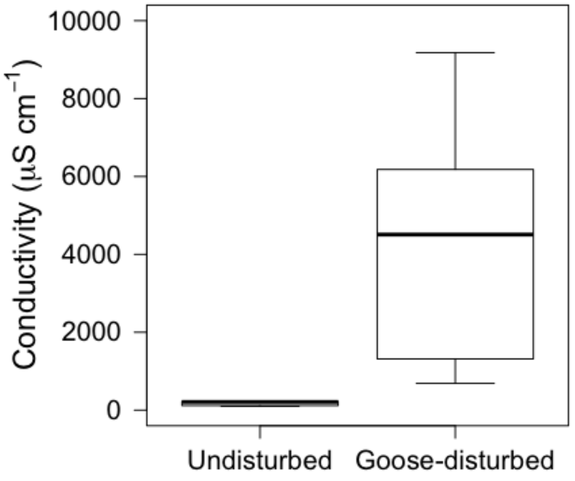

Figure S1. (A) The conductivity of enclosures throughout the experiments. Control enclosures are represented by the thin solid line. +Nutrient enclosures are represented by the dotted-dashed line. +Salt enclosures are represented by the solid thick line. +Nutrients+salt enclosures are represented by the dashed line. Vertical error bars are standard errors of the mean. Vertical dashed lines represent the 3 times that dispersers were added **(**B)The conductivity of ponds that are in the undamaged area (Undisturbed; n=5) and the ponds in the goose-damaged regions (Goose-disturbed; n=5)
